# Comparative Analysis of the Antioxidant Activity of *Cassia fistula* Extracts

**DOI:** 10.1155/2012/157125

**Published:** 2012-09-25

**Authors:** Md. Irshad, Md. Zafaryab, Man Singh, M. Moshahid A. Rizvi

**Affiliations:** ^1^Department of Biosciences, Jamia Millia Islamia, New Delhi 110025, India; ^2^School of Chemical Sciences, Central University of Gujarat, Gujarat, Gandhinagar 382030, India; ^3^Department of Medical Education, College of Medicine, King Saud University, Riyadh 11321, Saudi Arabia

## Abstract

Antioxidant potential of various extracts of *Cassia fistula* was determined by the DPPH, FRAP, Fe^3+^ reducing power, and hydrogen peroxide scavenging assay. Methanolic extracts of *Cassia fistula* showed the highest amount of phenolic and flavonoid content and reducing capacity, whereas hexane extracts exhibited the lowest level of reducing capacity. The order of antioxidant activity in *Cassia fistula* extracts displayed from higher to lower level as methanolic extracts of pulp, methanolic extracts of seed, hexane extracts of pulp, and hexane extracts of seed. The antioxidant potential of *Cassia fistula* extracts significantly correlated (*P* < 0.02) with the phenolic content of the methanolic extracts. Ascorbic acid taken as control showed highest antioxidant power in the present study.

## 1. Introduction 

*Cassia fistula *Linn. (*Caesalpiniaceae*) has great therapeutic implication in Indian system of medicine and exerts an antipyretic, analgesic, antiinflammatory, and hypoglycemic effects [1, 2]. Over the past few years, there has been an exponential growth in study of pharmacological properties of this plant [3–5]. Antioxidant components are microconstituents that inhibit lipid oxidation by inhibiting the initiation or propagation of oxidizing chain reactions, and are also involved in scavenging of free radicals. Clinical approaches of antioxidants increased multifold during the recent time for the management and therapeutic implication of neurodegenerative disorders, aging, and chronic degenerative diseases. In view of the above, we designed the study to evaluate the antioxidant potential and phenolic content in *Cassia fistula*.

## 2. Materials and Methods

### 2.1. Reagent and Chemicals

All the chemicals and solvents were of analytical grade and obtained from Merck and HiMedia, Mumbai, India. 

### 2.2. Preparation of *Cassia fistula* Extracts

The fresh ripe fruits of *Cassia fistula* were collected in June from the campus of Delhi College of Pharmaceutical Science and Research (DIPSAR), New Delhi, India, and properly authenticated. A voucher of specimen (PM 21) was stored in the laboratory for further reference. The fruit pulp and seed were separated and grounded to coarse powder. It was extracted with the hexane for 72 hrs and the same materials were reextracted with methanol for 72 hrs in soxhlet apparatus. The extract was filtered and dried in rotavapour. Hence, we obtained hexane extract of seed (HES), hexane extract of pulp (HEP), methanolic extract of seed (MES), and methanolic extract of pulp (MEP). 

### 2.3. Estimation of Total Phenolic Content

The total phenol content was estimated in the methanolic extracts of seed and pulp using Folin-Ciocalteu reagent (FCR) according to the procedure reported by Singleton et al. [6], using standard Gallic acid (*Y* = 1.8478*x* + 0.0702,  *R* ^2^ = 0.9971) and Tannic acid (*Y* = 1.388*x* + 0.0472,  *R* ^2^ = 0.9958), curve standardized in the lab for the calculation of Gallic acid equivalent (GAE) and Tannic acid equivalent (TAE) per gram of extracts, respectively. The blue complex was formed by the reduction of reagent by phenolic compounds in extract. Briefly, aliquot of 400 *μ*L of extract was added to 1.6 mL of sodium carbonate (7.5% in deionised water) and 2 mL of Folin-Ciocalteu reagent (diluted 10-fold in deionised water). After incubation of 1 hr at room temperature, absorbance was measured at 525 nm using LaboMed Inc. Spectrophotometer (USA). All determination was carried out in triplicate. Total phenolic content was expressed in mg GAE and TAE per gram of extracts, using calibration curve. 

### 2.4. Estimation of Flavonoid Content

Total flavonoids contents were estimated both in methanolic extracts of pulp and seed by method of Zhishen et al. [7], using Quercetin standard. Briefly, 0.5 mL of aliquot of extract was added to 75 *μ*L of 5% NaNO_2_ solution. After 6 minutes, 150 *μ*L of a 10% AlCl_3_ 6H_2_O solution was added and the mixture was allowed to stand another 5 minutes. Then, 0.5 mL of 1 mol/l NaOH and 2.5 mL of distilled water was added. The solutions were mixed and absorbance was measured at 510 nm using LaboMed Inc. Spectrophotometer (USA). All experiments were carried out in triplicate. Total flavonoid content was calculated as mgQE/g, using the following equation based on the calibration curve:

(1) $$\begin{array}{c} Y=2.234x+0.992,\;\;R^{2}=0.9927, \end{array}$$

where *x* was the absorbance and *y* was the mgQE/g.

### 2.5. DPPH Radicals Scavenging Activity

DPPH radicals scavenging of *Cassia fistula *extracts was estimated according to the method of Miliauskas [8, 9]. DPPH radicals absorbed maximum at 515 nm, which disappears with reduction by an antioxidant compound (s). Three milliliter (3 mL) DPPH solution in methanol (0.1 mM) was mixed with 100 *μ*L of extracts (mg/mL). In control 100 *μ*L methanol (without extracts) mixed with DPPH solution. The samples were incubated in a water bath for 20 min at 37°C, and the decrease in absorbance at 515 nm was measured. Ascorbic acid was used as positive reference. The experiment was carried out in triplicate. Radical scavenging activity was calculated using the following formula:

(2) $$\begin{array}{c} \%\;\text{inhibition}=\left[\frac{\left(\mathrm{AC}\;-\text{AE}\right)}{\text{AB}}\right]\times 100, \end{array}$$

where AC  = absorbance of the control and AE = absorbance of tested samples.

### 2.6. Ferric Reducing Antioxidant Potential (FRAP) Assay

Ferric reducing power of *Cassia fistula* extracts were determined using FRAP assay [10, 11]. This method is based on the reduction of colourless ferric complex (Fe^3+^ tripyridyltriazine) to blue-colored ferrous complex (Fe^2+^ tripyridyltriazine) by the action of electron donating antioxidants at low pH. The reduction was monitored by measuring the change of absorbance at 593 nm. The working FRAP reagent was prepared by mixing 10 volumes of 300 mM acetate buffer, pH 3.6, with 1 volume of 10 mM TPTZ (2,4,6-tri(2-pyridyl)-*s*-triazine) in 40 mM HCl and with 1 volume of 20 mM ferric chloride. All the required solutions were freshly prepared before their uses. 100 *μ*L of samples (mg/mL) were added to 3 mL of prepared FRAP reagent. The reaction mixture was incubated in a water bath for 30 min at 37°C. Then, the absorbance of the samples was measured at 593 nm. The difference between absorbance of sample and the absorbance of blank was calculated and used to calculate the FRAP value. FRAP value was expressed in terms of mmol Fe^2+^/g of sample using ferric chloride standard curve *Y* = 1.7057*x* − 0.2211,  *R* ^2^ = 0.9904. All measurements were calculated from the value obtained from triplicate assays.

### 2.7. Hydroxyl Radical Scavenging Activity Assay

The scavenging activity for hydroxyl radicals was measured with Fenton reaction [12, 13]. Reaction mixture contained 60 *μ*L of 1.0 mM FeCl_3_, 90 *μ*L of 1 mM 1,10-phenanthroline, 2.4 mL of 0.2 M phosphate buffer (pH 7.8), 150 *μ*L of 0.17 M H_2_O_2_, and 1.5 mL of extract at various concentrations. After incubation at room temperature for 5 min, the absorbance of reaction mixture was noted at 560 nm. The hydroxyl radicals scavenging activity was calculated according to the following equation and compared with ascorbic acid as standard:

(3) $$\begin{array}{c} \%\;\text{Inhibition}=\left(\frac{\left(\text{AB}-\text{AE}\right)}{\text{AB}}\times 100\right), \end{array}$$

where AB was the absorbance of blank (without extract) and AE was the absorbance of tested samples. 

### 2.8. Reducing Power Assay

The Fe^3+^ reducing power of extracts was determined by the method of Oyaizu [9, 11, 14]. The extract (0.75 mL) of various concentrations was mixed with 0.75 mL of phosphate buffer (0.2 M, pH 6.6) and 0.75 mL of potassium hexacyanoferrate (K_3_Fe(CN)_6_) (1%, w/v), followed by incubation at 50°C in a water bath for 20 min. The reaction was stopped by adding 0.75 mL of trichloroacetic acid (TCA) solution (10%) and mixture centrifuged at 800 g for 10 min. 1.5 mL of the obtained supernatant was mixed with 1.5 mL of distilled water and 0.1 mL of ferric chloride (FeCl_3_) solution (0.1%, w/v) for 10 min. The absorbance of reaction mixture was taken at 700 nm. Higher value absorbance of the reaction mixture indicated greater reducing power. 

## 3. Statistical Analysis

Results were expressed as mean value ± SD (*n* = 3). Regression analysis was performed to calculate the dose-response relation between the extracts. Linear regression analysis was performed to find out the correlation coefficient. Statistical significance was evaluated employing *t*-test and *P* < 0.05 which were considered to be significant.

## 4. Result and Discussion

### 4.1. DPPH Radical Scavenging

**Scheme 1:**
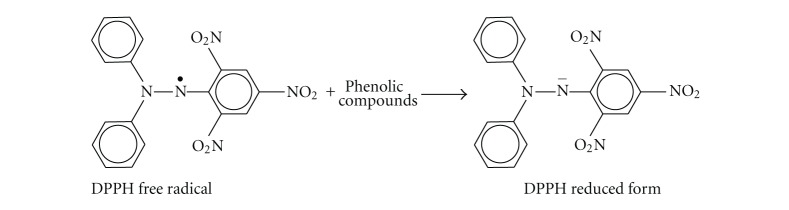


DPPH (1,1-diphenyl-2-picrylhydrazyl) analysis is one of the best-known, accurate, and frequently employed methods for evaluating antioxidant activity [15]. It is a stable free radical because of its spare electron delocalization over the whole molecule. The donation of H^+^ to the DPPH radicals made a corresponding change from violet colour to pale yellow in the solution. The DPPH scavenging also made a proportionate decrease in its absorbance at 517 nm (see Scheme 1). 

**Figure 1:**
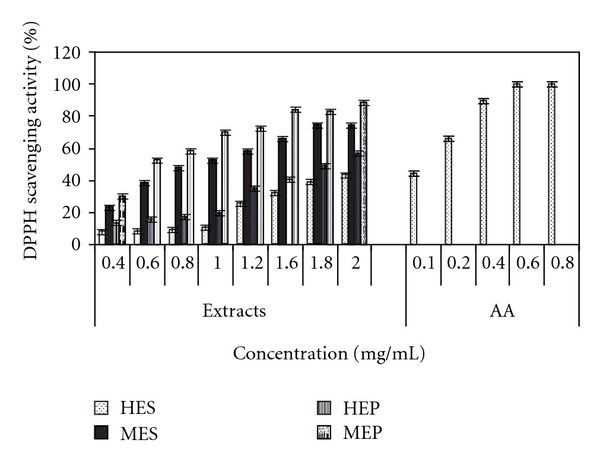
The graphical representation of DPPH scavenging activity of hexane extracts of seed (HES), pulp (HEP), methanolic extracts of seed (MES), and pulp (MEP). Values are expressed as mean ± standard deviation (*n* = 3). Ascorbic acid (AA) was used as a standard.

The order of DPPH scavenging against *Cassia fistula* extract was found to be in the order of *MEP* >MES > HEP > HES. Antioxidant activity of the methanol extracts of pulp and seed was also compared to total phenolic content and it was further found that radical scavenging effects of extracts were directly proportional to the phenolic content present in extracts (Table 1). Hence, methanol extract of pulp and seed showed the significant radical scavenging as the concentration increased, whereas hexane extract of pulp and seed showed low scavenging effect up to 1 mg/mL; thereafter, DPPH scavenging increased with the concentration of extract. The positive control, ascorbic acid showed maximum scavenging effect at very low concentration (Figure 1). A significant correlation coefficient (r, 0.978) was found between the antioxidant activity of the methanolic extract of pulp and seed. The proton radical scavenging action is known to be one of the important mechanisms for measuring antioxidant activity. This assay determines the scavenging of stable radical species *DPPH* by antioxidants compounds present in the extracts. The results showed the greater rate of DPPH scavenging activity of both the methanolic extracts as compared to thehexane extracts probably due to the presence of high content of phenolic compounds. EC_50_ value was determined from the plotted graph of scavenging activity against various concentrations of extracts, which is defined as the efficient concentration of antioxidant necessary to decrease the initial DPPH radicals concentration by 50% (Table 2). The lowest EC_50_ indicates the strongest ability of the extracts to act as DPPH radicals scavengers. Out of the all extracts, methanolic extract of pulp and seed showed the lowest EC_50_, 0.915 and 1.088 mg/mL, whereas hexane extracts of pulp and seed showed 1.865 and 2.239 mg/mL, respectively (Table 2). Ascorbic acid showed highest DPPH radicals scavenging with EC_50_ of 0.102 mg/mL. Sun and Ho reported a significant correlation between total phenolics and scavenging ability of buckwheat extracts on DPPH radicals [16]. Our study clearly indicated that the methanol extracts of pulp and seed of *Cassia fistula* exhibited high content of phenolic compounds which was significantly correlated with the DPPH radical scavenging activity (*P* < 0.005).

### 4.2. Hydroxyl Radical Scavenging Activity

The hydroxyl radicals are extremely reactive oxygen species that can react with every possible molecule in living organisms, especially with proteins, DNA, and lipids [17]. Hydroxyl radicals are capable of rapid initiation of the lipid peroxidation process by extracting hydrogen atoms from unsaturated fatty acids [18]. The electron or proton donation capacities of *Cassia fistula *extracts were further confirmed by the Fenton reaction system. Under *in vitro*, condition very reactive OH^−^ radicals were formed through the Fenton reaction. The reversible reaction between Fe^2+^ and 1,10-Phenathroline formed Fe(phen)_2_^2+^, which react with H_2_O_2_ and formed OH^−^ free radicals [19, 20]:

(4) Fe(phen)32+⇆Fe(phen)22++phen,Fe(phen)22++H2O2→Fe(phen)23++OH−+OH●.

**Figure 2:**
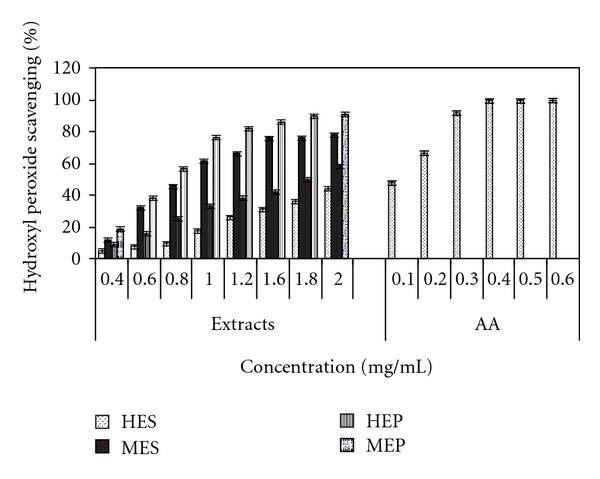
The graphical representation of hydroxyl radical scavenging activity of hexane extracts of seed (HES), pulp (HEP), methanolic extracts of seed (MES), and pulp (MEP). Values are expressed as mean ± standard deviation (*n* = 3). Ascorbic acid (AA) was used as a standard.

Among the reactive oxygen species (ROs), H_2_O_2_ is the most reactive and predominant radical generating molecule endogenously during aerobic metabolism [21]. The results showed that methanolic extracts of pulp and seed of *Cassia fistula* have scavenging ability of OH^−^ free radicals in a dose-dependent manner (Figure 2). The highest activity was noted for the pulp extract of methanol followed by methanolic extract of seed at all concentrations. OH^−^ free radical scavenging potential of all the extracts was found to be lower than that of the reference compound ascorbic acid. The EC_50_ values of MEP, MES, HES, HEP, and ascorbic acid were 0.889, 1.058, 2.075, 1.723, and 0.105 mg/mL, respectively (Table 2). The trend obtained in OH^−^ free radical scavenging behaviour was similar to the DPPH free radical scavenging nature (Figure 2). The result of OH^−^ free radical scavenging activity was significantly correlated with the DPPH free radical scavenging (*P*, 0.05). 

### 4.3. Reducing Power

**Figure 3:**
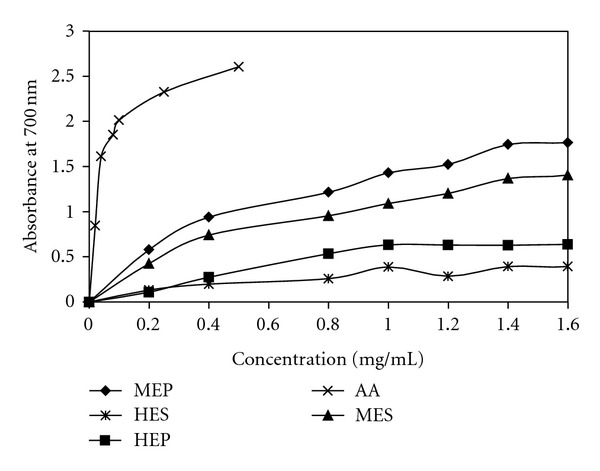
The graphical representation of reducing power of hexane extracts of seed (HES), pulp (HEP), methanolic extracts of seed (MES), and pulp (MEP). Values are expressed as mean ± standard deviation (*n* = 3). Ascorbic acid (AA) was used as a standard.

The reducing power assay is often used to evaluate the ability of an antioxidant to donate an electron [22]. In this assay, the ability of extracts to reduce Fe^3+^ to Fe^2+^ was determined. The presence of antioxidants in the extracts resulted into reduction of the ferric cyanide complex (Fe^3+^) to the ferrous cyanide form (Fe^2+^). In reducing power assay, antioxidants cause the reduction of the Fe^3+^ into Fe^2+^, thereby changing the solution into various shades from green to blue, depending on the reducing power of the compounds [23]. Strong reducing agents, however, formed Perl's Prussian blue colour and absorbed at 700 nm.  Figure 3 showed the reducing activities of various extracts of *Cassia fistula *in comparison with ascorbic acid as standard. The higher the absorbance of the reaction mixture, the higher would be the reducing power. Methanolic extract of pulp and seed showed some degree of electron donation. Reducing power of different extracts increased with the concentration of the extract. Hexane extract of seed and pulp showed less degree of Fe^3+^ reduction than the methanolic extracts. The reducing power was found to be in order of *MEP* >MES > HEP > HES. Interestingly, the rate of reducing power of methanolic extracts of both pulp and seed (MEP and MES) first increased rapidly with concentration but later it decreased. The reducing power of reference compound (Ascorbic acid) was found to be higher than all the tested extracts. It has been reported that the reducing power of substances is probably because of their hydrogen-donating ability [24]. Methanolic extracts of pulp and seed might, therefore, contain high amount of reductones than hexane extracts of pulp and seed. Hence, methanolic extracts of fruit pulp and seed may act as electron donors and could react with free radicals to convert them into more stable products and then terminate the free radical chain reactions.

Bhalodia et al. [25] recently reported antioxidant activity of the *Cassia fistula *flower. They reported that the hydroalcoholic extract of flower demonstrated significant radical scavenging activity in ferric reducing power and DPPH assays. It was also correlated with the total phenols which could be responsible for the antioxidant activity [25]. 

The reducing power assay is often used to evaluate the ability of an antioxidant to donate an electron which is an important mechanism of phenolic antioxidant action [17]. Many reports have revealed that there is a direct correlation between antioxidant activities and reducing power of certain plant extracts [26, 27].

### 4.4. FRAP Assay

FRAP assay measures the reducing potential of an antioxidant reacting with a ferric tripyridyltriazine (Fe^3+^-TPTZ) complex and producing a coloured ferrous tripyridyltriazine (Fe^2+^-TPTZ) [28]. The reducing properties associated with the presence of compounds exert their action by breaking the free radical chain through donating a hydrogen atom [29, 30]. FRAP assay showed positive correlation between reducing power and phenolic content in *Cassia fistula* extracts (Table 2). Here also, methanolic extracts of pulp showed greater FRAP value as 136.05 equivalent mmol of Fe^2+^/g sample. The other extracts MES, HEP, and HES showed FRAP value 112.02, 75.09, and 69.02 equivalent mmol of Fe^2+^/g samples, respectively. The result was similar with Benzie and Szeto [27], who found a strong correlation between total phenolic content and FRAP assay. Rice-Evans et al. [30] reported that phenolic compounds have redox properties, which allow them to act as reducing agents, hydrogen donators, and singlet oxygen quenchers. The redox potential of phenolic compounds played an important role in determining the antioxidant potential.

## 5. Conclusion 

Antioxidant potential of *Cassia fistula* extract demonstrated the highest reducing power in the methanolic extract of pulp and seed. It was concluded that the antioxidant activity of these extracts was directly proportional to the phenolic contents. The hexane extracts of seed and pulp, however, did not significantly reduce the free radicals under *in vitro* study.

## Figures and Tables

**Table 1:** Quantification of total phenolic and flavonoid contents in methanolic extract of pulp (MEP) and seed (MES).

*Cassia fistula* extracts	Total phenolic		
	Quercetine∗	Tannic acid∗∗	Flavonoids∗∗
MEP	114.23 ± 1.05	107 ± 0.87	74.65 ± 1.12
MES	109 ± 1.05	96 ± 1.08	67.78 ± 0.98

∗Total phenolic contents (mg gallic acid equivalent/g).

∗∗Total phenolic contents (mg tannic acid equivalent/g).

∗∗∗Flavonoid (mg quercetin equivalent/g).

**Table 2:** EC_50_ value is defined as the effective concentration of antioxidant necessary to decrease the radical concentration by 50%. FRAP values represent as equivalent mmol of Fe^2+^/gram sample.

*Cassia fistula* extracts	EC_50_ (DPPH) mg/mL	EC_50_ (hydroxyl peroxide) mg/mL	FRAP value
MEP	0.915	0.889	136.05
MES	1.088	1.058	112.02
HEP	1.865	1.723	75.09
HES	2.239	2.075	69.02
Ascorbic acid	0.102	0.105	—
